# *FAM20A* Mutations Can Cause Enamel-Renal Syndrome (ERS)

**DOI:** 10.1371/journal.pgen.1003302

**Published:** 2013-02-28

**Authors:** Shih-Kai Wang, Parissa Aref, Yuanyuan Hu, Rachel N. Milkovich, James P. Simmer, Mohammad El-Khateeb, Hinda Daggag, Zaid H. Baqain, Jan C-C. Hu

**Affiliations:** 1 Department of Biologic and Materials Sciences, University of Michigan School of Dentistry, Ann Arbor, Michigan, United States of America; 2 Oral Health Sciences Program, University of Michigan School of Dentistry, Ann Arbor, Michigan, United States of America; 3 Tehran University of Medical Sciences, Tehran, Iran; 4 Molecular Genetics Laboratory, National Center for Diabetes, Endocrinology, and Genetics (NCDEG), Amman, Jordan; 5 Faculty of Dentistry, The University of Jordan, Amman, Jordan; University of Pennsylvania, United States of America

## Abstract

Enamel-renal syndrome (ERS) is an autosomal recessive disorder characterized by severe enamel hypoplasia, failed tooth eruption, intrapulpal calcifications, enlarged gingiva, and nephrocalcinosis. Recently, mutations in *FAM20A* were reported to cause amelogenesis imperfecta and gingival fibromatosis syndrome (AIGFS), which closely resembles ERS except for the renal calcifications. We characterized three families with AIGFS and identified, in each case, recessive *FAM20A* mutations: family 1 (c.992G>A; g.63853G>A; p.Gly331Asp), family 2 (c.720-2A>G; g.62232A>G; p.Gln241_Arg271del), and family 3 (c.406C>T; g.50213C>T; p.Arg136* and c.1432C>T; g.68284C>T; p.Arg478*). Significantly, a kidney ultrasound of the family 2 proband revealed nephrocalcinosis, revising the diagnosis from AIGFS to ERS. By characterizing teeth extracted from the family 3 proband, we demonstrated that *FAM20A* ^−/−^ molars lacked true enamel, showed extensive crown and root resorption, hypercementosis, and partial replacement of resorbed mineral with bone or coalesced mineral spheres. Supported by the observation of severe ectopic calcifications in the kidneys of *Fam20a* null mice, we conclude that FAM20A, which has a kinase homology domain and localizes to the Golgi, is a putative Golgi kinase that plays a significant role in the regulation of biomineralization processes, and that mutations in *FAM20A* cause both AIGFS and ERS.

## Author Summary

*FAM20A* belongs to a family of 3 genes (*FAM20A*, *FAM20B*, and *FAM20C*) that encode kinases (phosphorylating enzymes) that modify proteins within the secretory pathway. FAM20C phosphorylates secretory calcium-binding phosphoproteins (SCPPs) that are critical for bone, dentin, and enamel biomineralization, and other calcium-binding proteins in milk and saliva. The function of FAM20A is unknown, but defects in the *FAM20A* gene have recently been shown to cause dental enamel defects along with enlarged gingiva (amelogenesis imperfecta and gingival fibromatosis syndrome or AIGFS; OMIM #614253). We identified three families with disease-causing mutations in *FAM20A*. All of the symptoms of AIGFS are also found in enamel-renal syndrome (ERS, OMIM #204690), which in addition features kidney calcifications known as nephrocalcinosis. We were only able to acquire a kidney ultrasound from one of our patients with *FAM20A* mutations, and it showed these kidney calcifications. We conclude that *FAM20A* mutations cause ERS and that persons diagnosed with AIGFS should have their kidneys examined. We also were able to obtain teeth from a patient with defined *FAM20A* mutations and to characterize the unusual mineral deposits that replace and add to normal tooth structures and may provide clues to the function of FAM20A.

## Introduction

Enamel-Renal Syndrome (ERS; OMIM #204690) is a recessive syndrome characterized by severely hypoplastic (thin) or aplastic enamel on both the primary and secondary dentitions, pulp stones, and failed or delayed eruption of much of the permanent dentition, particularly the posterior teeth. Coronal dentin is sometimes resorbed and replaced by lamellar bone and there is often hypercementosis on root surfaces. These dental symptoms are associated with nephrocalcinosis, although blood chemistry analyses are typically normal [1]–[3]. Gingival enlargement is sometimes noted [4], [5]. The initial patient complaint is the lack of enamel and failed eruption of many permanent teeth. Nephrocalcinosis is typically discovered by a renal ultrasound scan ordered because of the known association between this rare pattern of dental defects and renal dysfunction, rather than due to a patient complaint or history of renal problems [4]–[6]. In the original report of ERS one of the two affected individuals died of renal failure [1]. Another report described the results of a series of renal evaluations on an ERS patient that observed minimal renal calcifications at age 5 that became progressively denser in roentgenograms taken at ages 8, 11, and 14 years, and then stabilized [2]. Subsequent reports found the kidney calcifications in patients with ERS to be benign [3], [4], [7].

The literature describes patterns of recessive tooth defects similar to that observed in enamel renal syndrome (ERS), but without evidence of nephrocalcinosis [8]–[23]. As in ERS, the patient's initial chief complaint relates to the lack of enamel and failure of permanent tooth eruption. Dental radiographs reveal that most if not all of the teeth lack an enamel layer and have extensive pulp calcifications. The unerupted teeth show pericoronal radiolucencies delimited by a sclerotic margin. The teeth are usually smaller than normal, often with misshapened roots [12]. A common observation on radiographs is resorption of the occlusal surface (sometimes all the way to the pulp) of unerupted teeth [20]. When the malformed teeth are characterized histologically, they lack dental enamel, but show normal-looking dentin with well-formed dentinal tubules [13], [14]. The minimal “enamel” has no prismatic structure. On some teeth there is extensive localized root and/or crown resorption with partial replacement of the resorbed dentin by lamellar bone or in some places by globular structures comprised of incompletely coalesced concentric calcifications [12]. The thin roots are often covered by an abnormally thick layer of what appears to be cellular cementum [11], [19].

Recently, advanced genetic methods involving targeted exome capture, next generation DNA sequencing, and bioinformatics computer analyses implicated *FAM20A* (family with sequence similarity 20, member A) located on chromosome 17q24.2 as the defective gene in a recessive disorder manifesting the same oral features as described above [22], [24] and designated “Amelogenesis Imperfecta and Gingival Fibromatosis Syndrome” (AIGFS; OMIM #614253). The association between *FAM20A* and a syndrome that included severe enamel hypoplasia and gingival hypertrophy was confirmed by mutational analyses in four additional families that identified three homozygous *FAM20A* mutations (c.34_35delCT; c.813-2A>G; c.1175_1179delGGCTC) and compound heterozygous mutations (c.590-2A>G with c.826C>T) in four families [25]. In none of the families with *FAM20A* mutations were teeth available for microscopic examination or were renal ultrasounds performed.

We have characterized three families with a recessive syndrome caused by *FAM20A* mutations. All affected individuals in these families had mutations in both *FAM20A* alleles. Extracted molars were characterized histologically and shown to have hypercementosis and dentin replaced by lamellar bone. All findings were consistent with a diagnosis of AIGFS; however, we were intrigued by the similarity of AIGFS with ERS and inquired further about whether or not our probands had kidney problems.

## Results

### Family 1

**Figure 1:**
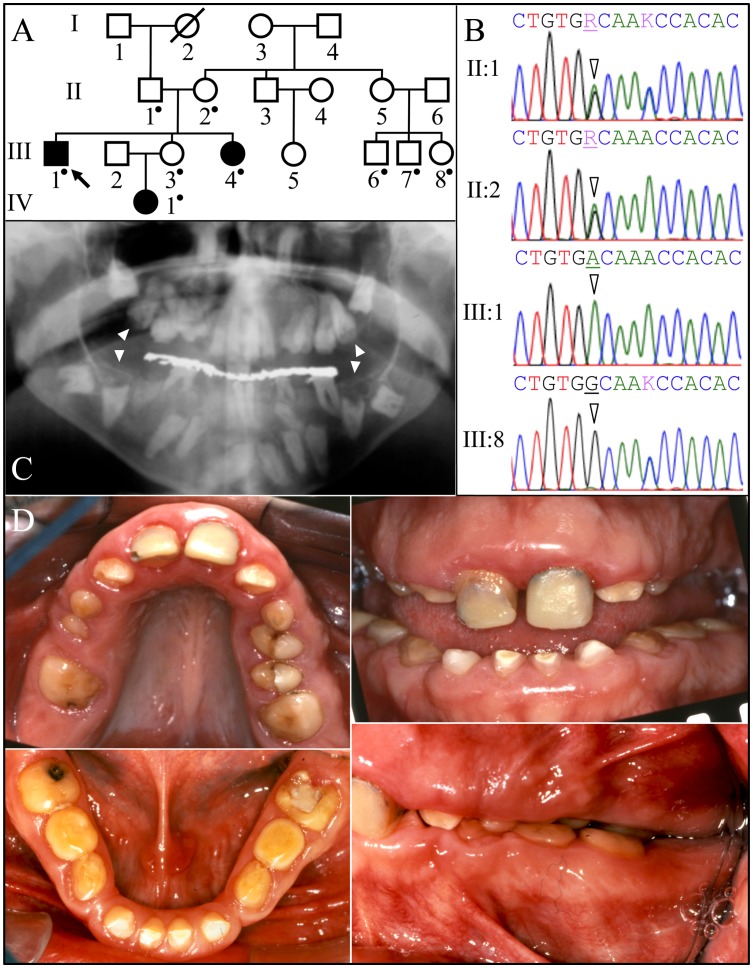
Family 1 from the Caribbean with *FAM20A* mutation c.992G>A; g.63853G>A; p.G331D. *A:* Pedigree. A dot marks person who donated samples for DNA sequencing. *B: FAM20A* exon 7 DNA sequencing chromatograms. The proband's parents (II:1 and II:2) were both heterozygous (R = A or G) at cDNA position 992 (arrowheads). The proband (III-1) had the c.992G>A transition mutation in both alleles of *FAM20A*. This mutation changed a conserved glycine with an aspartic acid (p.G331D). The proband's affected younger sister (III-4) and her infant niece (IV:1) were also homozygous for this mutation (not shown). II:1 and III:8 were heterozygous for a recognized polymorphism (rs2302234) in exon 7 (K = A or C) unrelated to the phenotype. *C:* Proband's panoramic radiograph. Note the many unerupted teeth. The mandibular and maxillary unerupted second molars show concave occlusal surfaces without enamel (arrowheads). *D:* Proband's oral photos. The maxillary central incisors are restored. The clinical crowns were short with hypoplastic enamel. There was a deep anterior overbite, a posterior cross-bite, and retained mandibular primary molars (letters K, L, S, T).

The proband's parents were born in the Caribbean. Oral photos and a panoramic radiograph were obtained for the proband and DNA was collected from nine family members (Figure 1). The proband (III:1), his younger sister (III:4), and niece (IV:1) were affected. According to the proband, when his adult teeth finally came in they were the same size as his baby teeth and very short. As far as he remembers, his gums have always been large and bumpy. He reported that he was otherwise healthy with average height and weight. Intraoral photographs of the proband showed a mixed dentition, small dental crowns with generally thin enamel, and yellow discoloration. Over-retained primary molars in the mandibular arch and partially erupted maxillary premolars were observed. The proband had a deep anterior overbite, a posterior cross-bite, and a class III molar relationship. The vertical dimension appeared to be reduced. Radiographically, the proband had the full complement of permanent teeth, but eruption of the canines, mandibular premolars and molars was failed or delayed. No radiopaque enamel layer was apparent on any of the teeth. Even unerupted teeth with completed root formation lacked enamel. Pericoronal radiolucencies outlined by sclerotic borders were observed around unerupted teeth, symptomatic of a slow expansion of the dental follicle covering the crown. In some cases the dentin occlusal surface appeared concave and close to the pulp chamber, suggesting pre-eruptive crown resorption. Most teeth showed intrapulpal calcifications. The gingiva appeared to be hyperplastic.

Family 1 was one of the original 24 AI families that we recruited for genetic studies [26]. No disease-causing mutations in the proband's DNA were identified during mutational analyses of the proven AI candidate genes (*AMELX*, *ENAM*, *FAM83H*, *WDR72*, *KLK4*, and *MMP20*) and *AMBN* [27]. *FAM20A* analyses however identified a G to A transition resulting in a missense mutation in exon 7 (c992G>A; g.63853G>A; p.Gly331Asp) that is homozygous in the proband (III:1), his sister (III:4) and niece (IV:1), heterozygous in proband's parents (II:1 and II:2) and unaffected sister (III:3), and absent from his three first cousins (III:6, III:7:, and III:8). This sequence variation has not been previously identified in the dbSNP database or in 1000 Genomes Project Pilot Data [28]. The glycine (G^331^) that is replaced by aspartic acid is conserved throughout vertebrate evolution (Figure S1) and the substitution was predicted to be probably damaging by PolyPhen-2 analyses [29]. This family was recruited almost 10 years ago and we have not been able to obtain any information concerning kidney calcifications.

### Family 2

**Figure 2:**
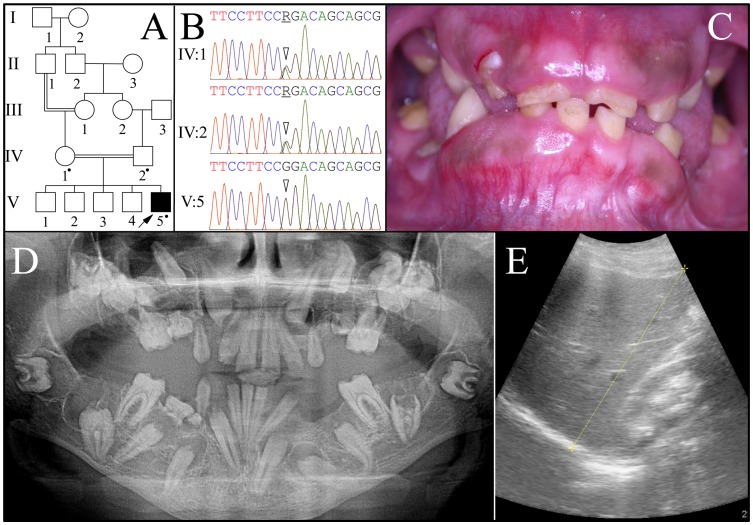
Family 2 from Jordan with *FAM20A* mutation c.720-2A>G; g.62232A>G; p.Q241_R271del. *A:* Pedigree: a dot marks person who donated samples for DNA sequencing. *B: FAM20A* intron 4 DNA sequencing chromatograms. The proband's parents (IV:1 and IV:2) were both heterozygous (R = A or G) at cDNA position 720 (2 arrowheads). The proband (V:5) had the c.720-2A>G transition mutation in both alleles of *FAM20A*. This mutation is predicted to cause the skipping of exon 5, which is predicted to delete 31 amino acids (Q241-R271) from the protein without shifting the reading frame. *C:* Proband's oral photo showing enamel hypoplasia, gingival enlargement and failed eruption. *D:* Proband's panoramic radiograph. Note the enamel hypoplasia, pulp calcifications, and unerupted teeth with pericoronal radiolucencies delimited by sclerotic borders. The left mandibular second molar (#18) shows apparent crown resorption. *E:* Ultrasound of proband's right kidney, located to the right of the yellow line.

Family 2 was a consanguineous family from Jordan (Figure 2). A panorex radiograph of the proband showed the retention of primary teeth and delayed eruption of permanent cuspids, premolars, and second molars. No radiopaque enamel was detected and expanded peri-coronal radiolucencies were evident on all unerupted teeth. The pulp chambers were typically calcified and nearer the occlusal surface than expected. On some unerupted teeth the crown occlusal to the pulp chambers had disappeared, as if by resorption. An ultrasound of the proband's kidneys revealed bilateral medullary nephrosis with small calcifications in both kidneys causing acoustic shadowing. Otherwise both kidneys were normal in size and corticomedullary differentiation, each measuring about 11 cm in bipolar length.

The proband (V:5) was the only affected person. *FAM20A* mutation analyses of the proband and his parents (IV:1 and IV:2) identified an A to G transition that altered the splice acceptor site at the end of intron 4 (c.720-2A>G; g.62232A>G) that is homozygous in the proband and heterozygous in both parents. This sequence variation has not been previously identified in the dbSNP database or in 1000 Genomes Project Pilot Data. Several possible aberrant RNA splicing outcomes could occur [30]. Skipping of exon 5 would delete 31 amino acids (p.Q241_R271del). No normal transcript variants skipping exon 5 are listed in GenBank. Retention of intron 4 would introduce a premature termination codon, and likely cause mutant *FAM20A* transcripts to be degraded by nonsense-mediated decay. Human Splice Finder version 2.4.1 [31] suggests there could be activation of a 5′ cryptic splice site that would add one nucleotide to exon 5 and lead to a frameshift and subsequent nonsense-mediated decay.

### Family 3

**Figure 3:**
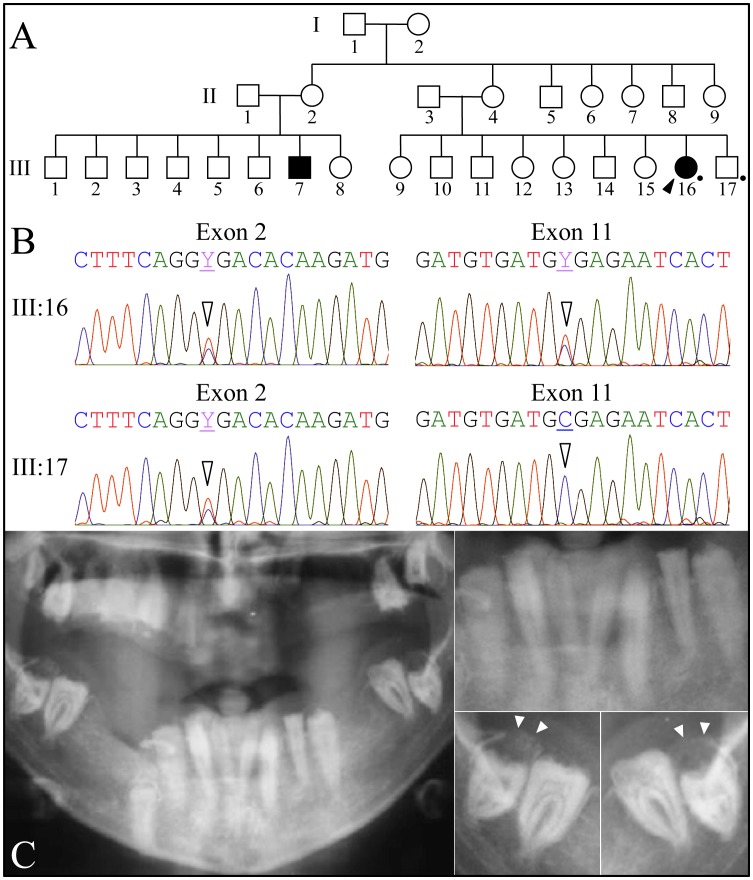
Family 3 from Iran with *FAM20A* nonsense mutations in exon 2 (c.406C>T; g.50213C>T; p.R136*) and in exon 11 (c.1432C>T; g.68284C>T; p.R478*). *A:* Pedigree consistent with a recessive pattern of inheritance. *B:* Exon 2 (left) and exon 11 DNA sequencing chromatograms. The proband (III:16) is heterozygous for nonsense mutations in exon 2 (c.406C>T) and exon 11 (c.1432C>T). The unaffected brother (III:17) is only heterozygous for the c.406C>T mutation in exon 2. *C:* Panoramic radiograph of proband. Note the lack of enamel, pericoronal radiolucencies over the unerupted mandibular third molars (arrowheads), and apparent crown resorption of the left mandibular second molar (#18).

Family 3 was a large kindred from Iran with two affected cousins (Figure 3). All of the proband's teeth were extracted (and some saved) prior to recruitment. A pre-surgical panoramic radiograph showed no radiopaque enamel, delayed tooth eruption, intrapulpal calcifications, and pericoronal radiolucencies. DNA was obtained from the proband and her younger, unaffected brother. Only *FAM20A* was characterized by mutational analyses. The proband was a compound heterozygote for two C>T transitions that both resulted in premature translation termination (TGA) codons. The nonsense mutations were in exon 2 (c.406C>T; g.50213C>T; p.R136*) and in exon 11 (c.1432C>T; g.68284C>T; p.R478*). The unaffected younger brother was heterozygous for the nonsense mutation in exon 2, but his exon 11 sequence was normal on both alleles. The exon 2 nonsense mutation was previously reported to cause AIGFS when found on both *FAM20A* alleles [24].

### Scanning Electron Microscopy

**Figure 4:**
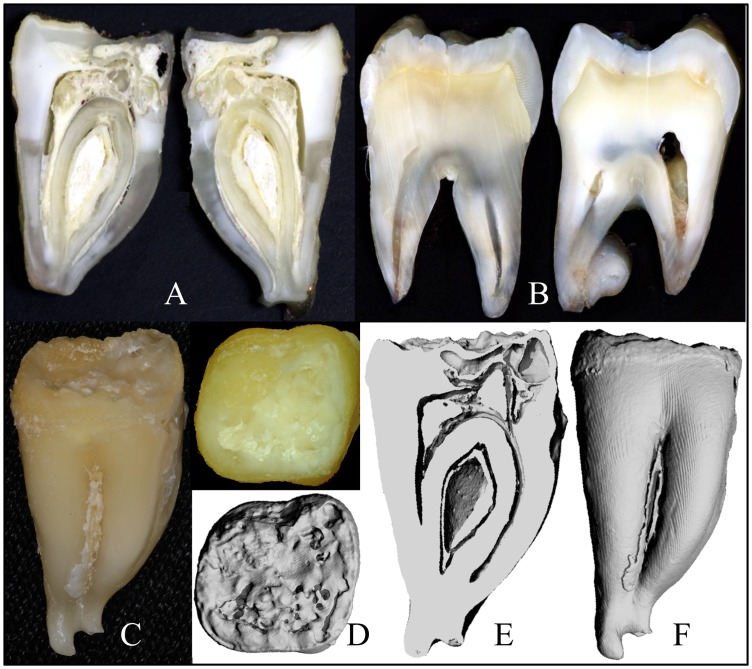
Images of *FAM20A* ^−/−^ tooth #18. *A:* Photographs of #18 after cutting it sagitally. *B:* Photographs of a wild-type molar after cutting it sagitally. *C:* Photograph of #18 before sectioning. *D:* Occlusal view of #18 by photograph (top) and 3-D μ-CT image. *E:* 3-D μ-CT image of inside #18. Note the hollow area in the crown and the calcified pulp chamber. *F:* 3-D μ-CT image of #18. Note the shortness of the crown, which as apparently greatly diminished by resorption.

Four extracted secondary teeth from the proband of family 3 were provided to us for analyses (Figure S2). The “enamel” on the crowns was thin, soft and crusty, and only evident near the cervical margins. The teeth were smaller than normal and showed irregularities of root form. The area coronal to the root furcation was sometimes expanded, while the roots themselves were short, thin, and sometimes fused. Some parts of the roots showed pronounced concavities resembling a row of bites from an apple. A mesial-distal cut was made through #18, the mandibular left second molar (Figure 4A, 4C) and compared to the normal tooth (Figure 4B). Much of the mesial half of the crown had been resorbed and was only partially replaced by mineral, producing “hollow” areas on 3-D μCT images (Figure 4D–4F), while much of the pulp chamber was calcified.

**Figure 5:**
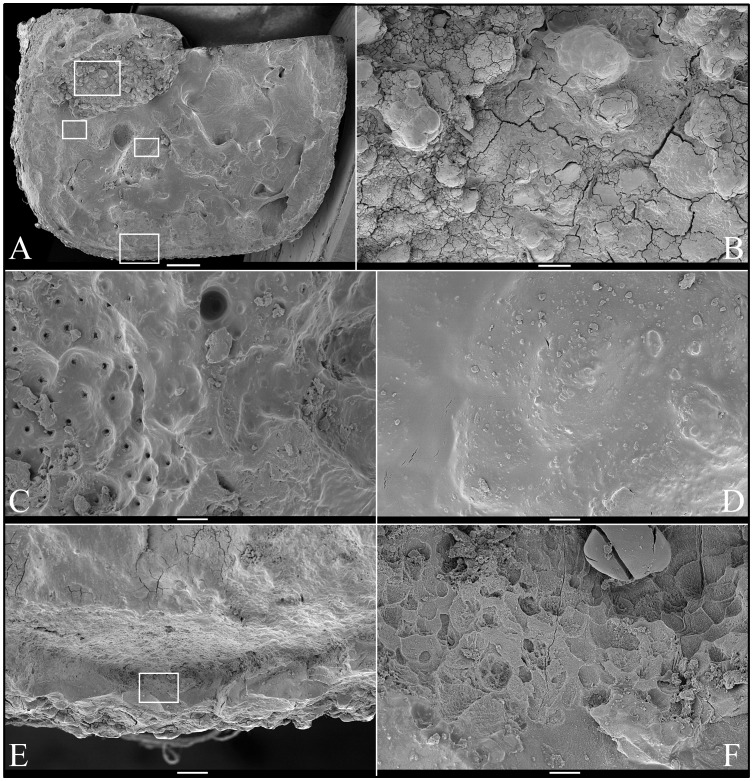
Scanning Electron Micrographs (SEMs) of molar (#18) occlusal surface. *A:* Low magnification view of occlusal surface after partially cutting and then splitting the tooth sagitally (mesial-distal direction) for SEM analyses (bar: 1 mm). The boxes, from top to bottom, are locations of higher magnification views shown in B–E, respectively. *B:* Region showing knob-like calcifications (bar: 100 µm). *C:* Region where dentinal tubules reach the surface (bar: 10 µm); *D:* Region showing a relatively smooth surface (bar: 10 µm). *E:* Region from edge of crown (bar: 100 µm); *F:* Higher magnification of box in panel E showing no true enamel and apparent resorption lacunae (bar: 10 µm).

**Figure 6:**
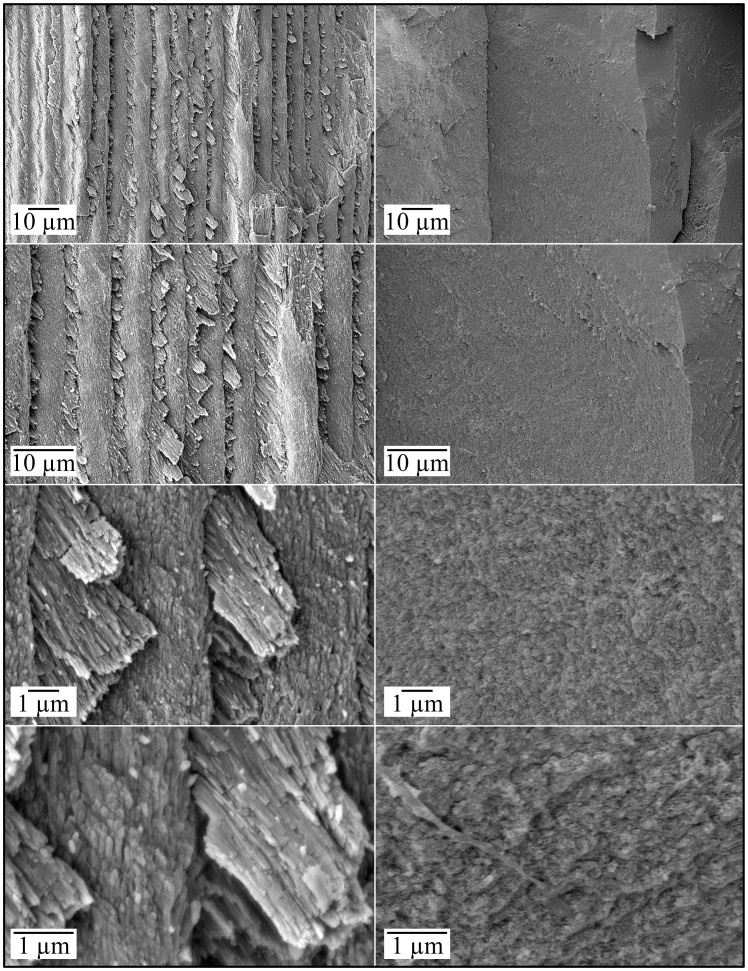
Scanning Electron Micrographs (SEMs) of mineral covering coronal dentin in a molar (#18) split for SEM examination. *Left:* Enamel layer in normal molar *Right:* Mineral covering dentin in *FAM20* ^−/−^ molar. No long thin crystals with rod/interrod organization are observed in the *FAM20* ^−/−^ molar.

**Figure 7:**
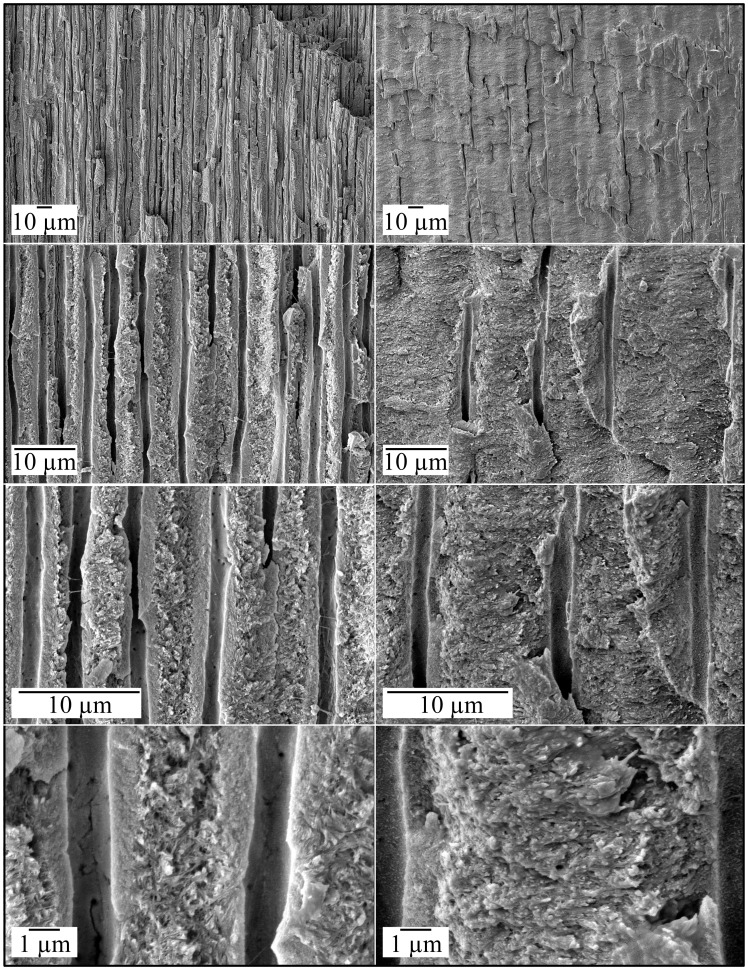
Scanning Electron Micrographs (SEMs) of dentin in a molar (#18) split for SEM examination. *Left:* Dentin in normal molar *Right:* Dentin in *FAM20* ^−/−^ molar. Dentin appears to be normal in the *FAM20* ^−/−^ molar.

**Figure 8:**
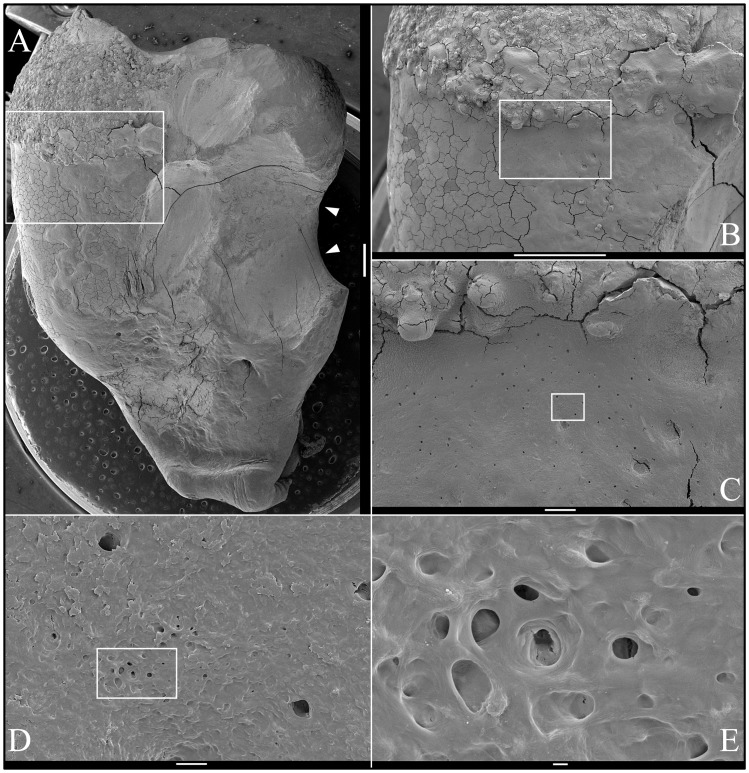
Scanning Electron Micrographs (SEMs) of molar (#32) showing root resorption. *A:* Mesial surface (bar: 1 mm). Large area of suspected root resorption (arrowheads). *B:* Higher magnification of region boxed in A (bar: 1 mm). Mineral covering dentin has knobby texture, while the root surface below the cervical margin appears to be smooth. *C:* Higher magnification of region boxed in B (bar: 100 µm). The apparently smooth root surface has surface craters and pits that look increasingly like resorption lacunae at higher magnification. *D:* Higher magnification of region boxed in C (bar: 10 µm). *E:* Higher magnification of region boxed in D (bar: 1 µm).

SEM analysis of the occlusal surface of the *FAM20A* ^−/−^ mandibular second molar (tooth #18; Figure 5A) revealed a variety of surface features, including rough, knob-like calcifications (Figure 5B), dentin with exposed dentinal tubules (Figure 5C), some relatively smooth mineral near the dentin surface (Figure 5D), and pitted “enamel” mineral superficially resembling volcanic rock on the lateral aspect of the crown (Figure 5E–5F). SEMs of deliberately fractured areas showed no mineral organization characteristic of true enamel (Figure 6). SEMs of dentin looked the same as normal dentin (Figure 7). SEMs of the mandibular third molar (tooth #32; Figure 8A) showed a relatively smooth root surface (Figure 8B) perforated by small holes (dentinal tubules) and larger craters suggestive of resorption lacunae (Figure 8C–8E).

### Backscatter Scanning Electron Microscopy

**Figure 9:**
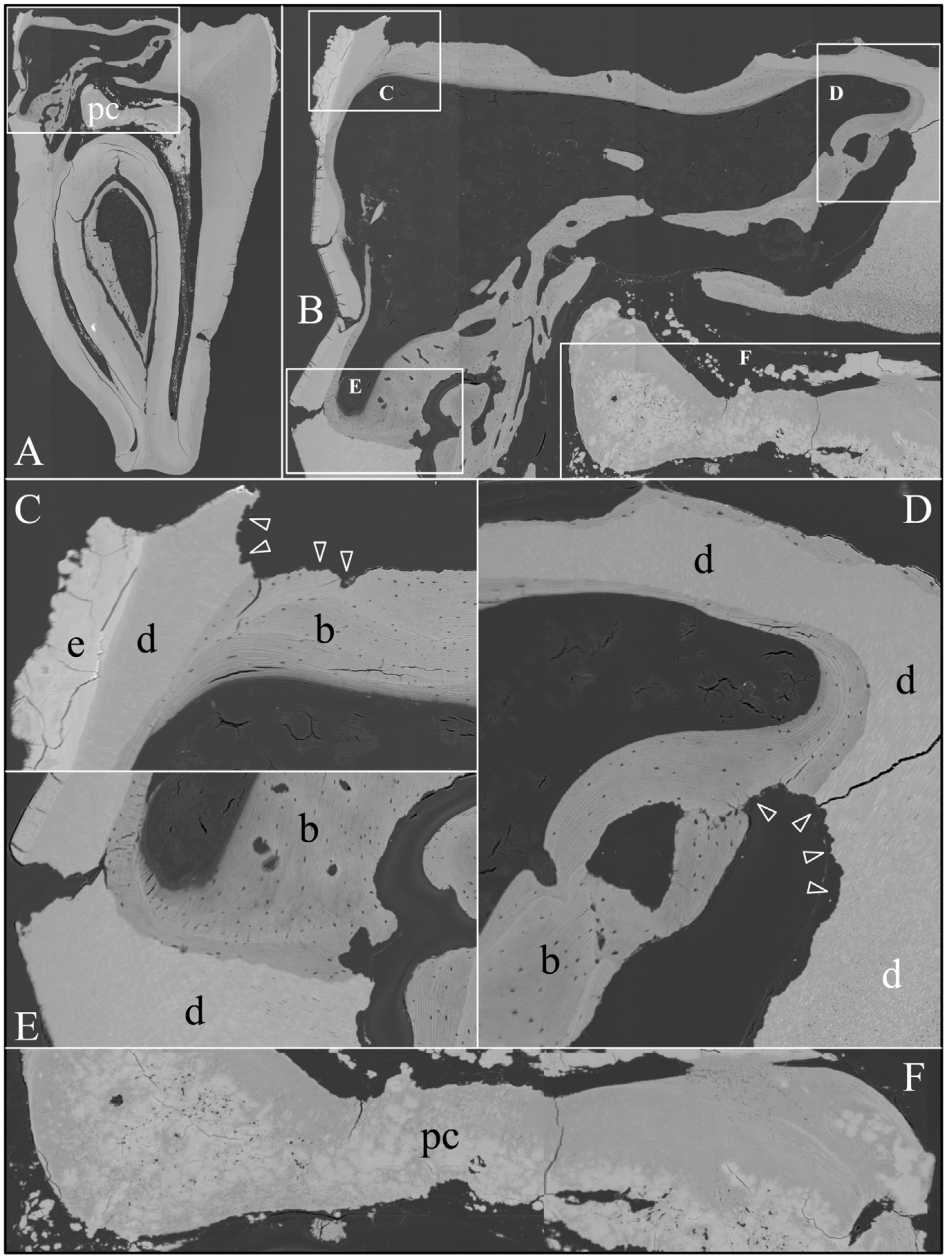
Backscatter Scanning Electron Micrographs (bSEMs) of molar (#18) crown. *A:* The bSEM of molar after it was cut sagitally (mesial-distally). *B:* Higher magnification of region boxed in A showing regions magnified in C–F. The bowtie-shaped structure in the lower right is the calcified pulp chamber. Most of the coronal dentin has been resorbed, with some of it replaced by well-formed lamellar bone (b). *C–E:* Region showing dense, rough, crusty mineral in place of enamel (e) covering sclerotic dentin (d) that is fused to lamellar bone (b). There appears to be sites of active resorption of the dentin and bone (arrowheads). *F:* The pulp calcification (pc) is comprised of coalesced spheres that resemble the crusty “enamel” in mineral density embedded in a second, less mineralized material like dentin or acellular cementum that lacks osteocyte lacunae.

**Figure 10:**
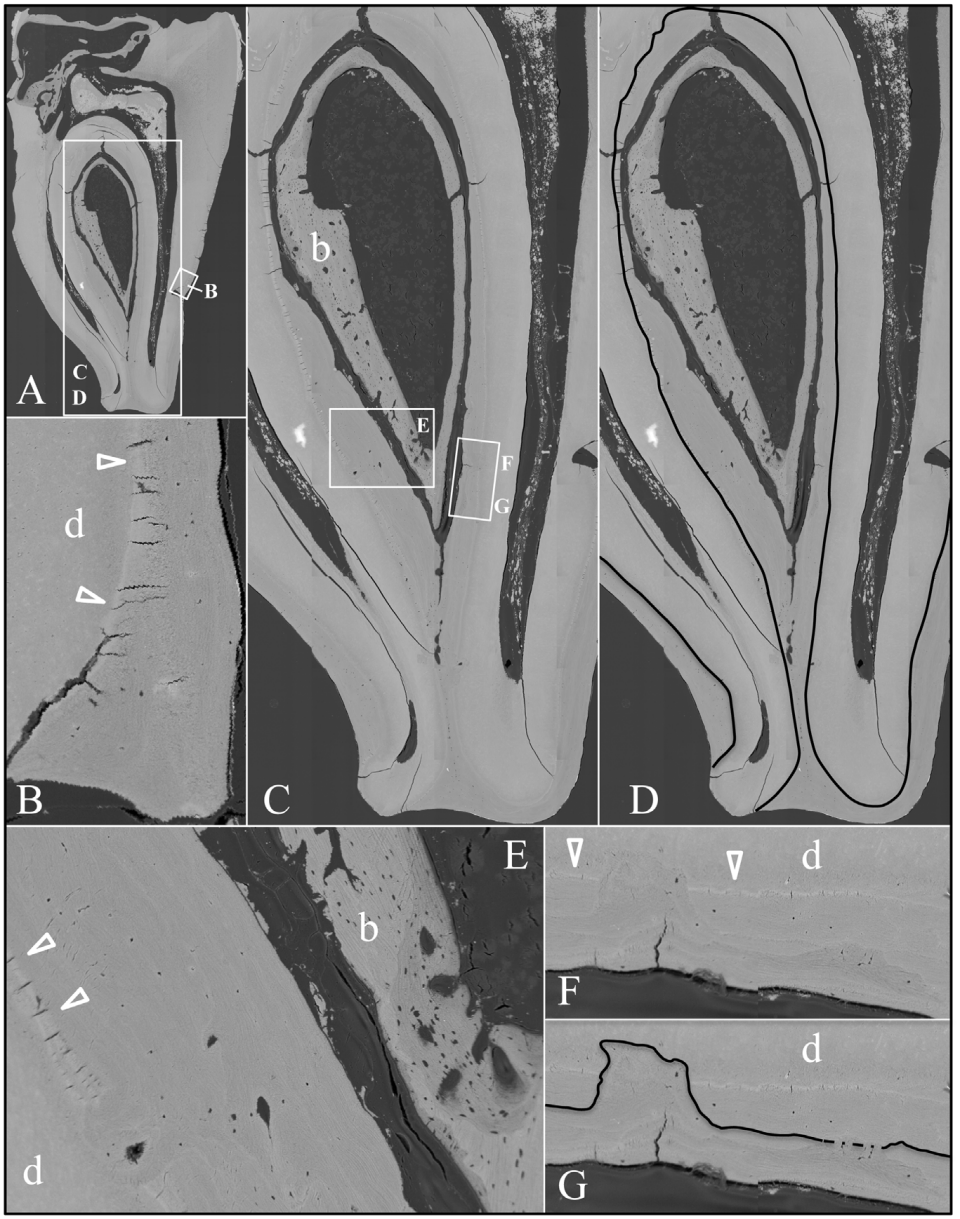
Backscatter Scanning Electron Micrographs (bSEMs) of molar (#18) roots. *A:* The bSEM of molar after it was cut sagitally (mesial-distally). *B:* Higher magnification of smaller box in A showing the layered build-up resembling cellular cementum. Arrowheads mark the dentin-cementum border. *C–D:* Higher magnifications of the larger box in A showing the thick layers of “cellular cementum” covering the roots. In panel D a dark line is placed at the dentin surface. *E:* Higher magnification of the larger box in panel C showing the thick layers of “cellular cementum” covering the roots and how the lamellar pattern suggests that deposition of these layers was punctuated by periods of resorption that sometimes penetrated into the dentin. *F–G:* Higher magnification of the smaller box in panel C also showing how deposition of the layers of acellular cementum was punctuated by resorption that sometimes penetrated into the dentin.

Backscatter SEMs of tooth #18 revealed that there was no enamel on the occlusal surface of the crown, although a thin, crusty material more highly mineralized than dentin covered part of the lateral coronal surfaces (Figure 9). Remarkably, only a small remnant of dentin, with apparent resorption lacunae at its edges, was evident in the mesial half of this *FAM20A* ^−/−^ molar crown. In its place was a laminated, bone-like material with osteocyte lacunae. Based upon the patterns of the growth lines, this lamellar bone had undergone repeated cycles of resorption and deposition. The calcifications in the pulp were unevenly mineralized. The most highly mineralized pulp material was comprised of incompletely coalesced spherical structures (calcospherites). Thus there appeared to be at least two types of pathological mineralization within the crown: lamellar-like bone and calcospherites that apparently formed by a different mechanism. Backscatter SEMs of the root region revealed thin roots, with normal-looking root dentin covered by a thick layer of laminated, cementum-like mineralized tissue similar to the lamellar mineralized tissue that had replaced dentin in the crown but with fewer osteocyte lacunae (Figure 10). The dentin was often separated from this thick cementum-like layer by a hypermineralized line that might represent the original cementum. This line was sometimes interupted at places where the root surface had been resorbed locally and replaced.

**Figure 11:**
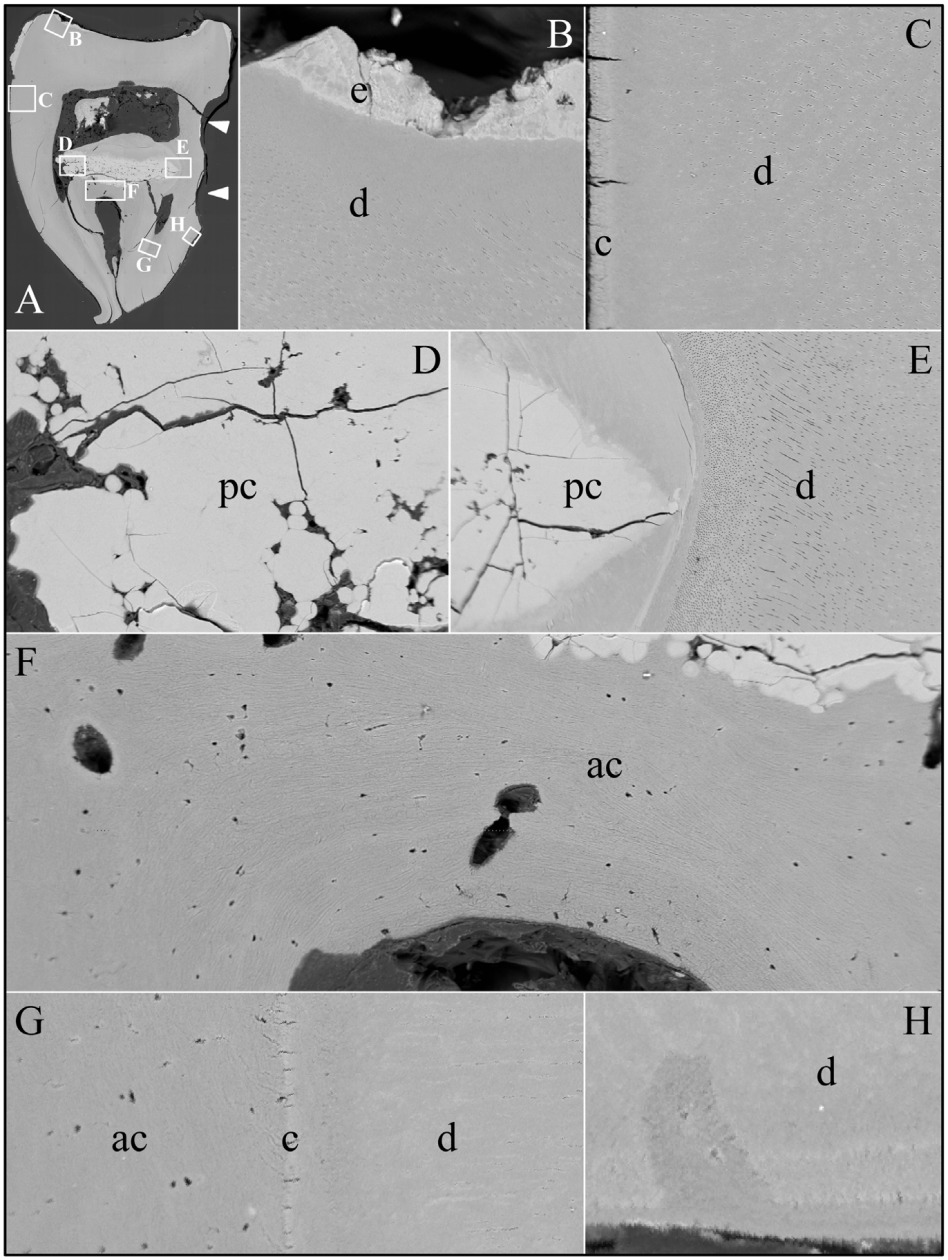
Backscatter Scanning Electron Micrographs (bSEMs) of molar (#32). *A:* The bSEM of molar after it was cut sagitally (mesial-distally). *B:* Rough “enamel” (e) covering sclerotic dentin. *C:* Acellular cementum covering sclerotic root dentin. *D–E:* Highly mineralized pulp or radicular calcifications (pc) comprised of coalesced spheres above the root furcation and associated with a less mineralized material that contacts dentin (d). *F:* The radicular area appears to be comprised entirely of acellular cementum (ac) or lamellar bone from the furcation to the highly mineralized coalesced spheres. *G:* Root dentin covered with a thick layer of acellular cementum (ac) or bone. A thin line of more highly mineralized material, possibly cementum (c), separates these layers. *H:* The material covering root dentin is deposited in layers and sometimes fills in areas of localized root resorption.

An unerupted third molar (#32) was also characterized by bSEM and showed a somewhat different pattern of pathological resorption and mineralization (Figure 11). The “enamel” layer was very thin, rough, and discontinuous. It was more highly mineralized than dentin in most places, and appeared to have coalesced from multiple mineral foci. The dentin had well-organized dentinal tubules. The pulp was partially calcified. Besides major resorption of dentin from part of the root surface, the bulk of the pathology was in the furcation area, which included a large hypermineralized region of coalesced calcospherites surrounded by lamellar bone that extended coronally to the pulp and apically beyond the furcation so that the bone-like material seems to have entirely replaced the dentin at the furcation but retained the original morphology of the furcation.

### FAM20A Localization to the Golgi

**Figure 12:**
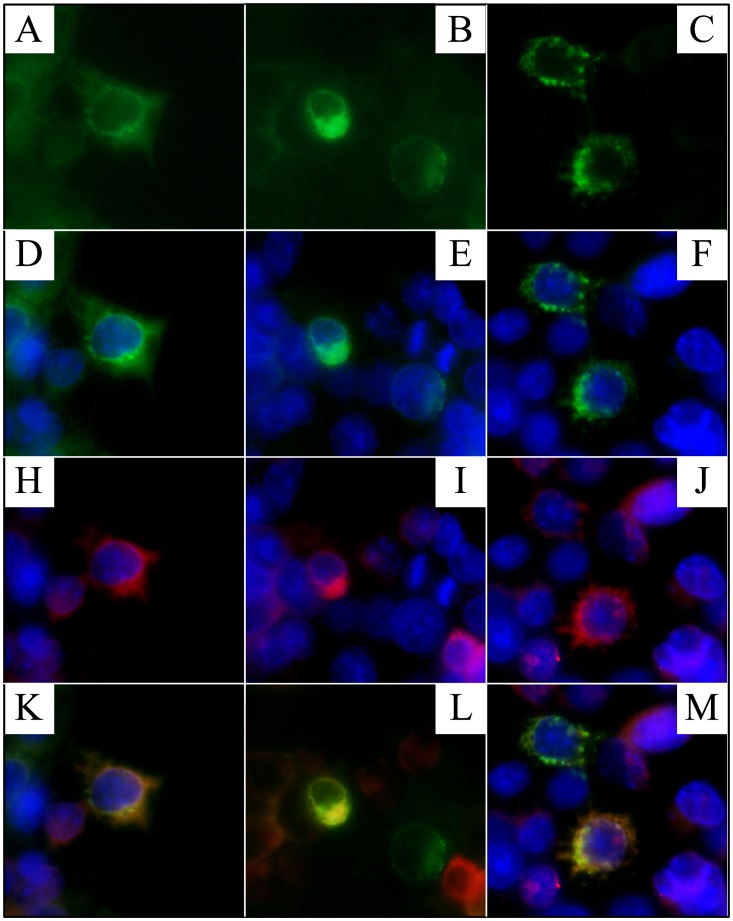
Localization of FAM20A to the Golgi. HEK293 cells were co-transfected with two plasmids. Cells that expressed the Golgi-GFP exhibited green fluorescence in the Golgi (A–F). FAM20A-flag was immunodetected and exhibited red fluorescence (H–J). Cells expressing both plasmids exhibited yellow fluorescence indicating superimposition of the two signals (K–M).

Human embryonic kidney (HEK) 293 cells were cotransfected with two plasmid constructs that expressed Flag-tagged FAM20A and a Golgi-GFP (green fluorescent protein) marker, respectively (Figure 12). In cells that received both constructs, the FAM20A signal superimposed upon that of the Golgi marker.

## Discussion

Amelogenesis imperfecta (AI) generally refers to non-syndromic forms of inherited enamel malformations that may include other local defects such as delayed tooth eruption and misshapened roots [32]. It is also used to describe this phenotype in syndromes. In the case of Enamel-Renal Syndrome (ERS) the dental problems may appear prior to any evidence of kidney disease and kidney ultrasounds are rarely performed on AI patients, so the condition can easily be misdiagnosed as autosomal recessive enamel agenesis (AI Type IG) [32]. This was the diagnosis we made years ago for the proband of family 1 [26]. We noted the mild gingival enlargement but did not consider the possibility of a kidney phenotype.

With the breakthrough discovery that *FAM20A* mutations cause AI associated with a variety of oral manifestations including eruption failures and gingival enlargement [24], we performed mutation analyses in three families exhibiting this pattern of defects, and in each case we identified disease-causing mutations in both *FAM20A* alleles and made the diagnosis of Amelogenesis Imperfecta and Gingival Fibromatosis Syndrome” (AIGFS). However, we read several reports showing the AIGFS pattern of oral manifestations in a person with kidney calcifications (nephrocalcinosis) [2]–[5], [33], [34]. In every case the nephrocalcinosis was discovered by radiographic or ultrasound images of the kidneys and not through a history of kidney problems in the family. Among the reports of similar cases without nephrocalcinosis, we found no cases in which a renal ultrasound was performed and found to be negative. After finally appreciating the kidney association, we were able to contact family 2 to obtain a kidney ultrasound, which detected nephrocalcinosis. The appropriate diagnosis for this family then is Enamel-Renal Syndrome (ERS). Unfortunately, we have been unable to obtain renal ultrasounds from affected individuals in families 1 and 3.

Additional support for the association of nephrocalcinosis and the absence of FAM20A comes from recent analyses of *Fam20a* null mice [35], which discovered that “two-thirds of *Fam20a* ^−/−^ mice had small kidneys with pitted surfaces, which showed widespread calcification…”. Another interesting finding was detecting *Fam20a* gene expression in the parathyroid gland, given the failure of posterior tooth eruption in AIGFS and ERS and that primary failure of tooth eruption (PFE, OMIM #125350) can be caused by mutations in *PTH1R* (OMIM 168468; 3p21.31), the gene encoding a receptor for both parathyroid hormone and parathyroid hormone-related protein [36].

### Scanning Electron Microscopy (SEM)

Our SEM analyses of *FAM20A* ^−/−^ molars detail an assortment of developmental malformations juxtaposed with secondary modifications. Developmentally, the enamel failed to form and the roots were small and misshapened. The crown and roots were susceptible to secondary resorption and turnover. Lamellar bone replaced parts of the resorbed crown, while a thick material resembling cellular cementum covered the roots. Highly mineralized, coalesced spherical calcifications were observed in the pulp and/or radicular area. We expect that the secondary root resorption and pathological mineralization occurred during the period of impaction. These unusual dental changes are rare in patients with non-syndromic amelogenesis imperfecta [27], [37], but are hallmark features of Enamel-Renal Syndrome (ERS) and were observed in the teeth from our probands with *FAM20A* mutations and nephrocalcinosis. These distinctive dental histological changes have previously been described in persons diagnosed as having AI without checking for nephrocalcinosis [8], [11], [12], [15]. The similarities between our histological observations and those reported for extracted teeth with a comparable dental phenotype are remarkable and increase our suspicion that ERS has been historically under-diagnosed.

*FAM20A* is part of a small gene family that in human and mouse has three members: *FAM20A*, *FAM20B*, and *FAM20C*. All three proteins have signal peptides of 21 amino acids and appear to be secreted [38]. The FAM20 genes encode proteins of similar size with a conserved C-terminal putative kinase domain (cd10469). *FAM20A* (17q24.2; cDNA reference sequence NM_017565.3) encodes a 541 amino acid protein. *FAM20B* (1q25; NM_014864.3) encodes 409 amino acids and *FAM20C* (7p22.3; NM_020223.2) 584 amino acids. Analysis of human expressed sequence tags (ESTs) suggests that the FAM20 family is expressed in many tissues. The National Center for Biotechnology Information (NCBI) human EST database currently has 5,779,625 entries for 45 healthy tissues. Among these are 103 EST entries for *FAM20A* (Hs.268874) from 22 tissues, with larynx (82/million), testes (60/million), and kidney (47/million) showing the highest proportion of *FAM20A* transcripts. Mice homozygous for a defined 58-kb deletion in the 5′ region of *Fam20a* showed growth-cessation and growth-delay [39]. Recently, *Fam20a* null mice were characterized with severe ectopic calcifications in the kidneys [35].

FAM20B is a xylose kinase in the Golgi required for the efficient addition of glycan attachments on secreted proteins. [40]. FAM20C has recently been identified as Golgi casein kinase [41], the enzyme that phosphorylates the secretory calcium binding phosphoproteins critical for biomineralization [42]. *FAM20C* mutations cause autosomal recessive lethal osteosclerotic bone dysplasia (Raine syndrome; OMIM #259775) [43], as well as non-lethal osteosclerotic bone dysplasia [44], [45]. FAM20A localizes to the Golgi, so perhaps FAM20A is a Golgi kinase like FAM20B and FAM20C, and its deficiency results in altered post-translational modifications of secreted proteins. In the absence of FAM20A, the dental follicle does not support tooth eruption, slowly expands, and generates psammomatous calcifications. The connective tissue of the gingiva also slowly expands and psammomatous calcifications are deposited within the hyperplastic gingiva [22]. Similar calcifications occur in the dental pulp and possibly in the kidneys, causing nephrocalcinosis. This pattern of ectopic mineralization might be explained by failure to catalyze appropriate post-translational modifications on extracellular matrix molecules that inhibit mineralization when FAM20A is absent.

The nine novel, disease-causing *FAM20A* mutations so far reported are obviously destructive of protein structure and function. Three are nonsense mutations (c.406C>T, p.Arg136*; c.826C>T, p.Arg276*; c.1432C>T, p.Arg478*). Three are splice junction mutations at the borders of exon 3 (c.590-2A>G, p.Asp197_Ile214delinsV), exon 5 (c.720-2A>G, p.Gln241_Arg271del) and exon 6 (c.813-2A>G, p.Arg271Serfs*70). Two are frameshifts (c.34_35delCT, pLeu12Alafs*67; c.1175_1179delGGCTC, p.Arg392Profs*22), and one is a missense mutation (c.992G>A, p.Gly331Asp) at a highly conserved site. Only one of the nine *FAM20A* disease-causing mutations was found in more than one family (c.406C>T). These data strongly implicate *FAM20A* in the etiology of this recessive disorder that combines enamel defects, retention of primary teeth, delayed and failed eruption of permanent teeth with pericoronal radiolucencies, pulp calcifications, small and misshapened teeth, gingival hyperplasia, and now, nephrocalcinosis. Only further studies can show if defects in other gene(s) can cause this pattern of malformations and if *FAM20A* defects were not previously associated with nephrocalcinosis due to a lack of penetrance, subclinical presentation, or delayed onset.

## Materials and Methods

The human study protocol and patient consents were reviewed and approved by the Institution Review Board at the University of Michigan and appropriate local Ethics Committees where families were recruited.

### DNA Isolation and PCR Amplification

Peripheral whole blood (5 cc) or buccal swabs were obtained from participating family members. Genomic DNA was isolated using the QIAamp DNA Blood Maxi Kit (Qiagen Inc, Valencia, CA) and (50 ng) from affected individuals was amplified using the Platinum PCR Supermix (Invitrogen, Carlsbad, CA), and the amplification products were purified using the QIAquick PCR Purification Kit (Invitrogen, Carlsbad, CA). The primer pairs and polymerase chain reaction conditions for the amplification of the coding regions were previously described for *AMBN* [26], *AMELX* [46], *ENAM* [47], *FAM83H* [48], *WDR72* [49], *KLK4* [50], and *MMP20* [51]. Twelve primer pairs were synthesized to amplify the eleven *FAM20A* exons (Figure S3), which covered all coding sequences and intron/exon borders. These *FAM20A* reactions were annealed at 57°C for 60 s, extended at 72°C for 90 s, and run for 35 cycles.

### Fabrication of Fam20a-Flag Expression Construct

A full-length mouse *Fam20a* cDNA clone (BC029169) in pCMV-SPORT6 was obtained from Thermo Scientific Open Biosystems (Lafayette, CO, USA). Restriction sites were introduced before the *Fam20a* translation initiation codon (NotI) and replacement of the translation termination codon (SalI) by PCR, which generated a 1645-bp amplicon (primer set: gcggCCGCTTGGGCCATGCCCG, agtcgacGCTCGTCAGATTAGCCTG). The amplification product was extracted from the gel and ligated into pCR2.1-TOPO (Invitrogen). The *Fam20a* coding region was excised by double digestion with NotI and SalI and ligated into pCMV-Tag 4 (Agilent), which had been restricted with NotI and SalI. Proper construction of the recombinant expression plasmid was verified by DNA sequencing.

### Cell Culture, Plasmid Transfection, and Immunocytochemistry

HEK293 cells were cultured with 2 mL Dulbecco's Modified Eagle Medium (DMEM) with 10% fetal bovine serum (FBS) in a Lab-Tek chamber slide (1 chamber) with cover (70360-12, Electron Microscopy Sciences, Hatfield, PA, USA) to reach 60% confluence on the day prior to transfection. Four µg of pCMV-Tag 4-Fam20a plasmid in 10 µL of Lipofectamine2000 (Invitrogen) was diluted with 500 µL of Opti-MEM® I reduced serum media (Invitrogen), and incubated for 20 min at room temperature. The pCMV-Tag 4-Fam20a/Lipofectamine2000 complexes were then added to the culture media. After 6 h, the culture media with complexes were replaced with 2 mL fresh media containing 20 µL of Golgi marker, CellLight Golgi-GFP BacMan 2.0 (C10592, Invitrogen). After 18 h, the cells were fixed with 4% paraformaldehyde for 15 min at room temperature, washed with PBS buffer 3 times, and then permeabilized with PBST for 15 min at room temperature. Following blocking with 5% sheep serum in PBST for 30 min at room temperature, anti-Flag antibody (1∶200, F7425, Sigma-Aldrich) was applied. After over-night incubation of primary antibody at 4°C, the cells were washed with PBS buffer for 15 min and then incubated for 30 min at room temperature in solutions containing anti-rabbit IgG secondary antibody conjugated with Alexa Fluor 594 (1∶500, A-11012, Invitrogen). The slides were then rinsed in PBS buffer for 15 min, mounted with ProLong Gold antifade reagent with DAPI (P-36931, Invitrogen), and examined under a Leica DM5000B fluorescence microscope.

### Micro-CT

The teeth were secured on petri-dish containing 1% agarose, scanned and analyzed using a SCANCO μCT-100 series micro-computed tomography system at the University of Michigan School of Dentistry micro-CT core.

### SEM

Tooth specimens were glued to a stub and sputter coated with gold for 75 s and then imaged using a Field Emission Gun Scanning Electron Microscope (FEG-SEM; Amray 1910 Field Emission Scanning Electron Microscope) at the Microscopy and Image Analysis Laboratory at the University of Michigan.

### Backscatter SEM

Teeth were cut using a slow diamond saw (Model 650, South Bay Technology, Inc, San Clemente, CA, USA), infiltrated by 1∶1, 1∶2 and 1∶3 acetone∶Epon for 12 h, degassed twice, infiltrated overnight with pure Epon, polymerized at 60°C for 48 h with the cut surface placed face down in a 25 mm SeriForm mounting cup (Struers, Ballerup, DK). The blocks were sequentially polished with successively finer grades (400, 800 and 1200) of silicone carbide paper (South Bay Technology, Inc) followed by 4 h of polishing with 1.0 micro alumina abrasive with Multitex Polishing Cloth using a Buehler Supermet 2 Position Polisher (Lake Bluff, IL), sonication and rinsed with water. The finely polished tooth surface was coated with carbon and imaged using the Cameca SX-100 Electron Microprobe Analyzer (CAMECA, 92622 Gennevilliers Cedex, FR) at the University of Michigan Electron Microbeam Analysis Laboratory (EMAL) using the backscatter mode at a beam current of 15 kV and 10 nA.

## Supporting Information

Figure S1 Sequence conservation at family 1 mutation site and *FAM20A* gene structure. *A:* Alignment of FAM20A protein sequences from human (*Homo sapiens*), mouse (*Mus musculus*), rat (*Rattus norvegicus*), chick (*Gallus gallus*), frog (*Xenopus tropicalis*), and fish (*Danio rerio*). The glycine (Gly331) changed to aspartic acid in family 1 is in bold. *B: FAM20A* gene structure diagram. Exons are shown in bold, with coding regions filled in white and non-coding region in gray. Introns are indicated by black lines (intron lengths are not drawn to scale). The locations of the four *FAM20A* mutations are marked a through d. *C: FAM20A* gene structure. Key: Length is the number of nucleotides in each segment; Gene is the range of nucleotides in the *FAM20A* genomic reference sequence (primary assembly; #NC_000017.10); cDNA (1) is the range of nucleotides in the *FAM20A* cDNA reference sequence (#NM_017565.3); cDNA (2) is the range of nucleotides in the *FAM20A* cDNA reference sequence (numbered starting from the translation initiation codon); # of aa is the number of amino acids encoded by each exon; and Range lists the amino acid segment encoded by each exon.
(TIF)

Figure S2 Photographs of four unerupted permanent teeth extracted from the proband of family 3. The tooth number and length of the tooth from cusp tip to root apex is provided below each tooth. Arrowheads mark large areas of apparent root resorption. *A:* Left mandibular first molar (#18). Radiographic images of this molar *in situ* are shown in Figure 3, micro-CT images in Figure 4, SEMs in Figure 5, Figure 6, and Figure 7, and backscatter SEMs in Figure 9 and Figure 10. *B:* Right mandibular third molar (#32). Radiographic images of this molar *in situ* are shown in Figure 3, SEMs in Figure 8, and backscatter SEMs in Figure 11. Note that the hypercementosis evident on the backscatter SEMs is not evident on the oral photographs or radiographs. *C:* Maxillary right third molar (#1). *D:* Mandibular left first bicuspid (#21).
(TIF)

Figure S3 Oligonucleotide primers used to amplify and then sequence *FAM20A* exons and adjoining intron sequences. The sizes of the amplification products are shown on the right.
(DOCX)
