# Supplementary figures and images for: FAM20A Mutations Can Cause Enamel-Renal Syndrome (ERS)
Source: PLoS Genet. 2013 Feb 28;9(2):e1003302. doi: 10.1371/journal.pgen.1003302 (PMC3585120; doi:10.1371/journal.pgen.1003302)

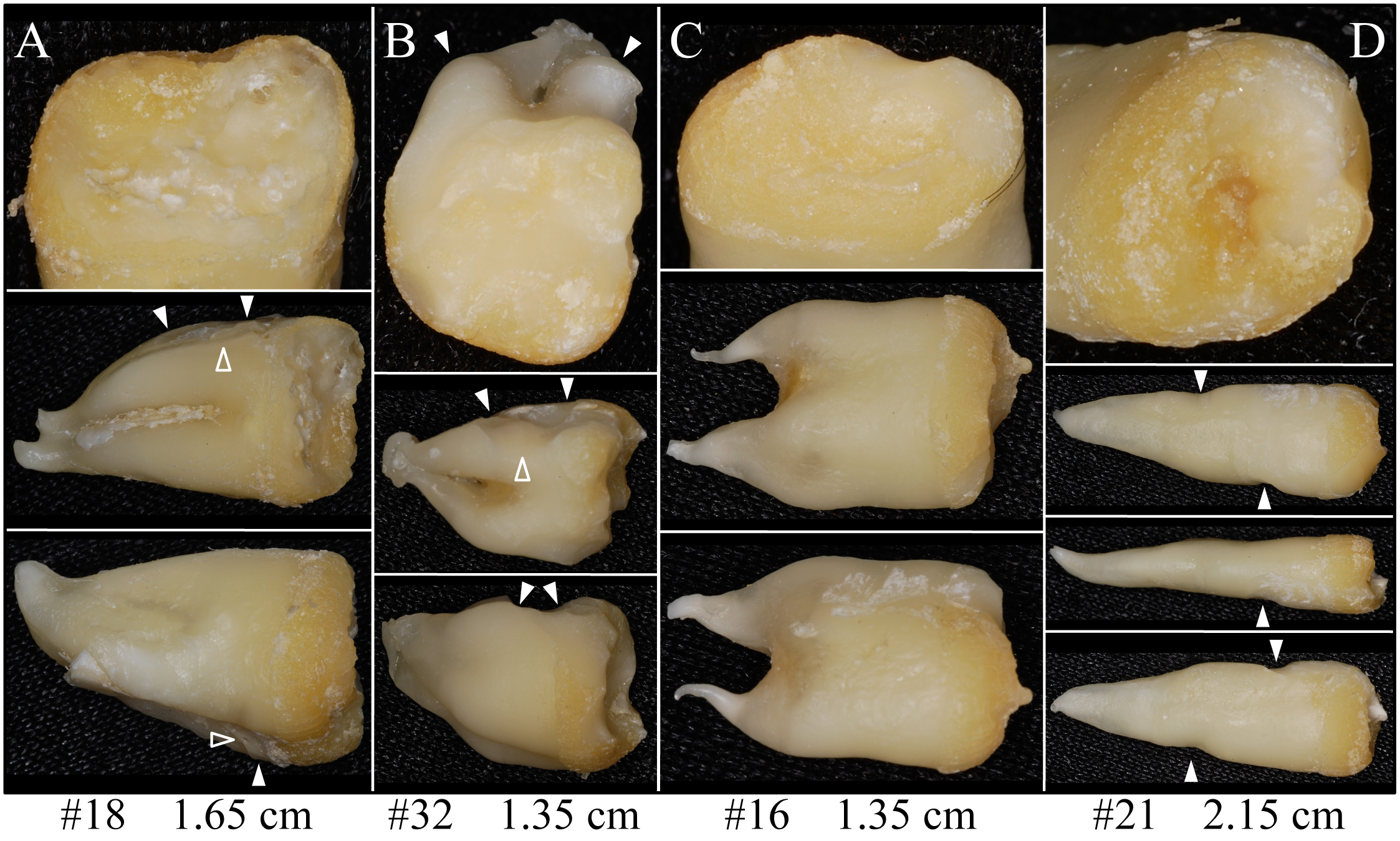

Supplement: Figure S2 — Photographs of four unerupted permanent teeth extracted from the proband of family 3. The tooth number and length of the tooth from cusp tip to root apex is provided below each tooth. Arrowheads mark large areas of apparent root resorption. A: Left mandibular first molar (#18). Radiographic images of this molar in situ are shown in Figure 3, micro-CT images in Figure 4, SEMs in Figure 5, Figure 6, and Figure 7, and backscatter SEMs in Figure 9 and Figure 10. B: Right mandibular third molar (#32). Radiographic images of this molar in situ are shown in Figure 3, SEMs in Figure 8, and backscatter SEMs in Figure 11. Note that the hypercementosis evident on the backscatter SEMs is not evident on the oral photographs or radiographs. C: Maxillary right third molar (#1). D: Mandibular left first bicuspid (#21). (TIF) [file pgen.1003302.s002.tif]
