# Supplementary material for: FAM20A Mutations Can Cause Enamel-Renal Syndrome (ERS)
Source: PLoS Genet. 2013 Feb 28;9(2):e1003302. doi: 10.1371/journal.pgen.1003302 (PMC3585120; doi:10.1371/journal.pgen.1003302)
Supplement: Figure S3 — Oligonucleotide primers used to amplify and then sequence FAM20A exons and adjoining intron sequences. The sizes of the amplification products are shown on the right. (DOCX) [file pgen.1003302.s003.docx]

**S3.** Primers used to amplify *FAM20A* exons.

| Primer | Oligonucleotide Sequence | Size (bp) |
| --- | --- | --- |
| Ex1-1F  Ex1-1R | 5'-CGACAGCATCCTCAGGAGAG  5'-CTCGGTTCGGGAAAAGTTGT | 682 |
| Ex1-2F  Ex1-2R | 5'-GACTCCAGGCACCTCTTCTG  5'-CTGGACTGTGAGGTCTGCAA | 689 |
| Ex2F  Ex2R | 5'-AAGGACCCCACAGGTGTTTT  5'-CCTCCTTATGGGGTCAGGAT | 563 |
| Ex3F  Ex3R | 5'-ACAGTGCCTGACTCGCAGTA  5'-ATGGTGGCATTTCAGAGACA | 412 |
| Ex4-2F  Ex4-2R | 5'-TACTGTCTTGAGCCCCTGGA  5'-AGTTCTGTGCTTGGGCAGAT | 417 |
| Ex5F  Ex5R | 5'-CTTGCAAGAAGCCTGTCTCC  5'-ACTAGAGCAACTGCCCCAAG | 572 |
| Ex6F  Ex6R | 5'-CAGGAGGCTTGACGGAATAA  5'-CACAACCTTTCCCATCCAGT | 548 |
| Ex7F  Ex7R | 5'-TCCCAGGAGGTTCAGCTTAG  5'-TATGAAATGGGGCAAAGGAG | 500 |
| Ex8F  Ex8R | 5'-ACGGAATATGCAGCCTTACG  5'-ATGAGTGACGACCCCTTGAG | 503 |
| Ex9F  Ex9R | 5'-TGAAGCCAGTCCATTTAGGG  5'-TCAAGTGGTTCTTGCAAAGG | 481 |
| Ex10F  Ex10R | 5'-GTGATCTGACTGGGGAGAGG  5'-GCAGTCTGTCATTGGCTGAG | 403 |
| Ex11F  Ex11R | 5'-GGCAAAGGACTGCAGGATAC  5'-CCTGCTTCCTTCCTAGCTGA | 586 |
